# Achieving excellent microformability in aluminum by engineering a unique ultrafine-grained microstructure

**DOI:** 10.1038/s41598-019-46957-4

**Published:** 2019-07-23

**Authors:** A. Dhal, S. K. Panigrahi, M. S. Shunmugam

**Affiliations:** Manufacturing Engineering Section, Department of Mechanical Engineering, Indian Institute of Technology Madras, Chennai, 600036 India

**Keywords:** Nanoscale materials, Mechanical engineering, Mechanical engineering, Nanoscale materials

## Abstract

During microforming of conventional materials, specimen and microstructural length-scales are close to each other. This leads to an abnormal deformation behavior of the material and reduces microformability. Engineering ultrafine-grained (UFG) microstructure in the material is a possible solution. However, micro-scale deformation behavior of UFG material is not fully understood. Present work attempts to comprehensively investigate the micro-scale deformation of four distinctly engineered microstructures: UFG with residual dislocations and elongated grains, UFG free of residual dislocation with equiaxed grains, bimodal-grained and coarse-grained. The deformation behavior is captured via micro-scale uniaxial tensile test and micro-deep drawing operation. Micro-cups generated from UFG material with equiaxed grains show excellent surface quality, form-accuracy and minimal process scatter. Postmortem microscopy of the formed micro-cups attributes this improved microformability to the activation of grain boundary-mediated plasticity in the material which results in synergetic grain migration and rotation. Presence of residual dislocations and elongated grains hinders the grain migration and rotation leading to strain localization and thinning. In case of bimodal and coarse-grained material, cross-slip based deformation mode progressively dominates over grain migration and rotation, which results in a reduction in microformability due to the influence of size-effect.

## Introduction

Driven by rising demand from the smartphone, smart-wearables, biomedical devices and avionics industries, the demand for micro-components is growing at an unprecedented rate. According to technical analysts, the annual turnover for industries manufacturing micro-electromechanical system alone is expected to reach USD 27 billion milestones by 2022^1^. Continuous research and technological development are going on to improve the quality and productivity of micro-components. Microforming emerges as an effective micro manufacturing process which can greatly complement the existing process to make quality micro-components. Microforming has the distinct advantages of high production capability, repeatability and effective material utilization. Despite its potential, large-scale industrial application of microforming is limited to date. The prime reasons behind this are the departure from conventional material deformation behavior and increase in process scatter, which make it difficult to control the quality of microformed parts. This phenomenon is termed as ‘size-effect’ and has been a subject of intensive research since the past two decades^2^.

The microstructure of a polycrystalline material typically consists of several grains whose average size varies from few to several hundreds of micrometers. As the sample size is miniaturized, the number of grains within the deformation zone is reduced. When the share of the number of grains in the deformation zone approaches unity, drastic changes in the material behavior are observed. These phenomena were captured using simple mechanical tests (e.g., uniaxial tensile tests, compression tests) by various researchers for a wide variety of materials, microstructures and process parameters. Commonly, four major phenomenological shifts in the material’s plastic behavior have been reported: (i) decrease in the yield strength of the material, (ii) greater scatter in the material strength and ductility, (iii) anisotropic material deformation behavior resulting in non-homogenous deformed component, and (iv) degradation of the surface morphology of the deformed samples^3^. Conventional rules of plasticity fail to explain the reason behind the above-mentioned behavioral abnormalities. Researchers have postulated that, due to the presence of a fewer number of grains within the deformation zone, the deformation behavior approaches that of the single-crystal material. This results in an increase in anisotropy and process scatter. Also, during miniaturization, the volume fraction of surface grains exceeds the volume fraction of core grains. Unlike core grains, which are surrounded by well-defined grain boundaries, free surfaces do not act as a reliable source for dislocation which result in a decrease in the material yield strength. Due to unequal strain accommodation capabilities of surface grains and core grains, strain localization occurs near their interface, which results in deterioration of the surface quality of the deformed components^4^.

A possible solution to tackle the size-effect is by increasing the number of grains in the deformation zone of the micro-samples. Although a substantial amount of research has been done to correlate the grain size with the microformability in the micrometer range, very few attempts have been done to explore the microformability of ultrafine-grained (UFG) or nanostructured materials. In UFG materials most of the grains are in the range of 100–1000 nm. These materials can be fabricated via severe plastic deformation (SPD) methods. UFG materials developed via SPD technique are suitable for macro/micro forming applications because of (i) lack of porosity, impurities and other imperfections in materials, (ii) ability to develop materials in bulk size in form of sheets, rods, plates, discs and, (iii) low processing costs^5^. One of the SPD techniques called cryorolling (CR) is particularly advantageous in terms of its yield, quality, and efficiency. Thin plates, sheets, and foils developed via CR can be used for microforming applications with relative ease. During CR, dynamic recovery due to adiabatic heating of the material is effectively suppressed resulting in accumulation of high dislocation density in the material in the form of tangles and forests. The extent of suppression in dynamic recovery depends on the stacking fault energy (SFE) of the material. In the case of high SFE materials such as Al (SFE = 166 mJ/mm^2^), cross-slip is activated when the level of strain imposed in the material is sufficiently high^6^. The microstructure of CR Al consists of thin nanometer-sized bands crisscrossed at 45° and 135° to the rolling direction which result in the formation of distorted rhombic nanostructured grains. Additionally, these grains possess substructures such as dense dislocation walls (in grain boundaries) and dislocation debris (in the grain interiors)^7^. These substructures act as pining points for accelerated strain localization resulting in premature failure during room temperature tensile loading^8^. Therefore, the uniform ductility and strain hardening ability of CR materials in tension are found to be extremely low rendering it unfit for any forming operations. However, by careful post-CR thermal treatment, it is possible to annihilate the excessive dislocations without a significant change in the grain size. Such annealed UFG microstructure has high strain hardening capabilities and uniform ductility and can be potentially used for microforming applications. Annealing also results in development of bimodal microstructure consisting of UFG and coarse grains which results in high ductility while retaining large tensile strength of the material^9^.

However, the microforming capability of materials with UFG and bimodal microstructure is rarely investigated. The first attempt to microform UFG material was made by Rosochowski and his collaborators in 2007^10^. They found greater uniformity in the shape and improved properties in the UFG micro-component compared to its coarse-grained counterpart. In their later work, they found reduced process scatter in UFG material compared to coarse-grained material^11,12^. Kim *et al*. developed high-quality micro-V grooves and pyramid pattern via superplastic microforming of UFG Mg-Al-Zn alloy^13^. Durst *et al*. successfully performed nano and micro-imprinting on the surface of nanocrystalline and UFG Ni^14^. In the recent times, significant contributions were made by Jie Xu’s research group to study the microforming capabilities of UFG Al, Cu and Mg-Li alloys^15–18^. Although excellent microformability has been reported for UFG materials, the governing microscale deformation mechanism of UFG materials has not been fully understood. Few attempts have been made to investigate the microscale deformation behavior and fracture mechanism of UFG materials by performing tension and compression tests of micro-samples and by examining the internal and external morphologies of fractured samples^19,20^. In one study, a transition from strain-hardening to strain softening mechanism was observed for UFG microstructure during micro-compression. It is also suggested that the shear-dominated fracture which is prevalent in UFG materials is more prominent in microscale tensile deformation. These types of investigations are essential to engineer desirable microstructure for process-productivity and product-quality enhancement during microforming. This knowledge bank can be further broadened to encompass microforming of various geometry, materials and strain paths. For this purpose, a systematic investigation of microstructure-microformability correlation is necessary. In the present work, a novel attempt has been made to study the microforming behavior of four distinct microstructures: (i) partially recovered UFG microstructure with slightly elongated grains and residual dislocations (UFG1), (ii) fully recovered UFG microstructure with equiaxed grains (UFG2) (iii) bimodal microstructure containing both ultrafine and coarse grains (BM), (iv) uniformly coarse-grained microstructure (CG). These four microstructures cover a wide range of grain size, morphology and distribution. This is also the first attempt to develop micro-components (in form of micro-cups) from thin sheets of a wide variety of microstructures by the micro-deep drawing process^21–24^ and compare their microformabilities.

## Methodology

### Development of sample with four different microstructures via SPD based thermomechanical process

For the present investigation, a commercially pure grade aluminum (AA1070) with a purity of 99.7% has been used. The material was procured in the form of 10 mm (t_o_) thick plates, which was subsequently cut into 50 × 50 mm square samples. These samples were heated in a closed furnace at a temperature of 400 °C for 1 hour, followed by water quenching. The heat-treated samples were submerged in liquid nitrogen bath for 15 minutes and cryorolled (CR) using a 2-high rolling mill fitted with rollers of 110 mm diameter and operating at a rolling speed of 8 rpm. After each pass, the sample thickness was measured, and the rolled sample was immersed back in the liquid nitrogen bath for 15 more minutes. Sample temperature was measured at the entry and exit of the CR process. Per pass rolling strain (ln(t/t_o_)) was limited to 0.25 and the CR was continued until a final sheet thickness of 500 μm (total rolling strain = 3.0) was obtained.

By using wire-electro-discharge machining, samples for uniaxial tensile test, microstructural characterization and microforming were cut from the 500 μm sheet. For the size-effect study, it is necessary to obtain sheets of various thickness keeping the microstructure of the material unchanged. To achieve this, mechanical polishing was done on a few CR samples using a series of fine to superfine emery sheets to reduce the sample thickness close to 300 μm and 100 μm from 500 μm. Four unique microstructures were obtained by annealing the CR samples (all three thicknesses) in the furnace for 200 °C, 225 °C, 250 °C for 30 minutes and 500 °C for 6 hours followed by quenching in cold water.

### Electron microscopy of the samples

Electron backscatter diffraction (EBSD) technique was used to capture the microstructure of all four samples chosen for the present investigation. For this purpose, FEI^®^ Inspect F50 field emission scanning electron microscope (SEM) was used (operating voltage = 20 kV, working distance = 15 mm, spot size = 5). To maximize the indexing efficiency, samples were meticulously cleaned and then electropolished using chilled Struers^®^ A2 solution at 10 V. The polishing time has been optimized based on several trial runs. EBSD scans were done at multiple sites throughout the sample cross section (plane between rolling-normal direction) to capture many grains. At least 500 grains were considered for statistically robust calculation of various microstructural parameters using EDAX^®^ TSL-OIM software. Very fine sub-granular details of some samples were obtained by using a 120 kV Philips^®^ CM12 transmission electron microscope (TEM). To obtain the required transparent sample for TEM study, a disc of 3 mm diameter was machined from the desired sheet. It was first mechanically polished to 90 μm using superfine emery papers, and then twin-jet electrolytic polishing was done using a solution of methanol, and perchloric acid (9:1) chilled to −40 °C at 5 V for 3 minutes.

### Uniaxial tensile test of the samples with varying microstructures and thicknesses

The uniaxial tensile test was performed in Instron^®^ 5584 universal testing machine fitted with a load cell of 2 kN (0.5% accuracy) and a 20 nm resolution linear encoder. Miniature tensile sample (gage length = 3 mm, gage width = 1 mm) was used to capture the tensile behavior of the samples. The miniature sample geometry was chosen to capture the size-effect associated with microforming. Samples of 500, 300 and 100 μm corresponding to all four microstructural conditions were selected for the tensile test. Before testing, samples were electropolished to develop a stress-free and smooth surface for tensile testing. The tests were performed at a crosshead speed of 1 mm/min till fracture. Five tensile samples were tested corresponding to each condition to ensure repeatability of results.

### Micro-deep drawing experiments

To perform the microforming experiment, a micro-deep drawing tool was custom designed and used. The mating tolerance of the press tool components is limited to 5 μm to obtain high accuracy of the formed micro-cups. An inverted configuration with the punch at bottom half is adopted to prevent deflections and improve stability during forming. Punch and die used in the micro-deep drawing tool have a diameter of 1.5 mm and 2.0 mm respectively with a corner radius of 0.5 mm. The blank diameter was optimized by several trials of limiting drawing ratio experiments using circular blanks of various diameters. Finally, a blank of 3 mm diameter (drawing ratio = 2) and 250 µm thickness was chosen for the present investigation. The forming was done at a ram speed of 1 mm/min, and the micro-forming was carried out until the micro-cup was fully deformed. For each case, at least ten micro-cups were fabricated, and five samples were selected for further analysis.

### Form, surface and cross-sectional examination of the micro-cups

Surface appearance and accuracy of the shape and size of the formed micro-cups was examined using SEM imaging technique. For cross-section examination, the samples were mounted in a conductive cold-setting mold (Technovit^®^ 5000), sliced using a low-speed wafer cutter, polished and studied under SEM. Cross section measurement was done at an interval of  0.05 mm from bottom-center to the flange. Finally, EBSD scanning was carried out at three locations (bottom, corner and wall) of the component cross-section.

## Results

### Characterization of the four microstructures and quantification of key microstructural parameters

**Figure 1:**
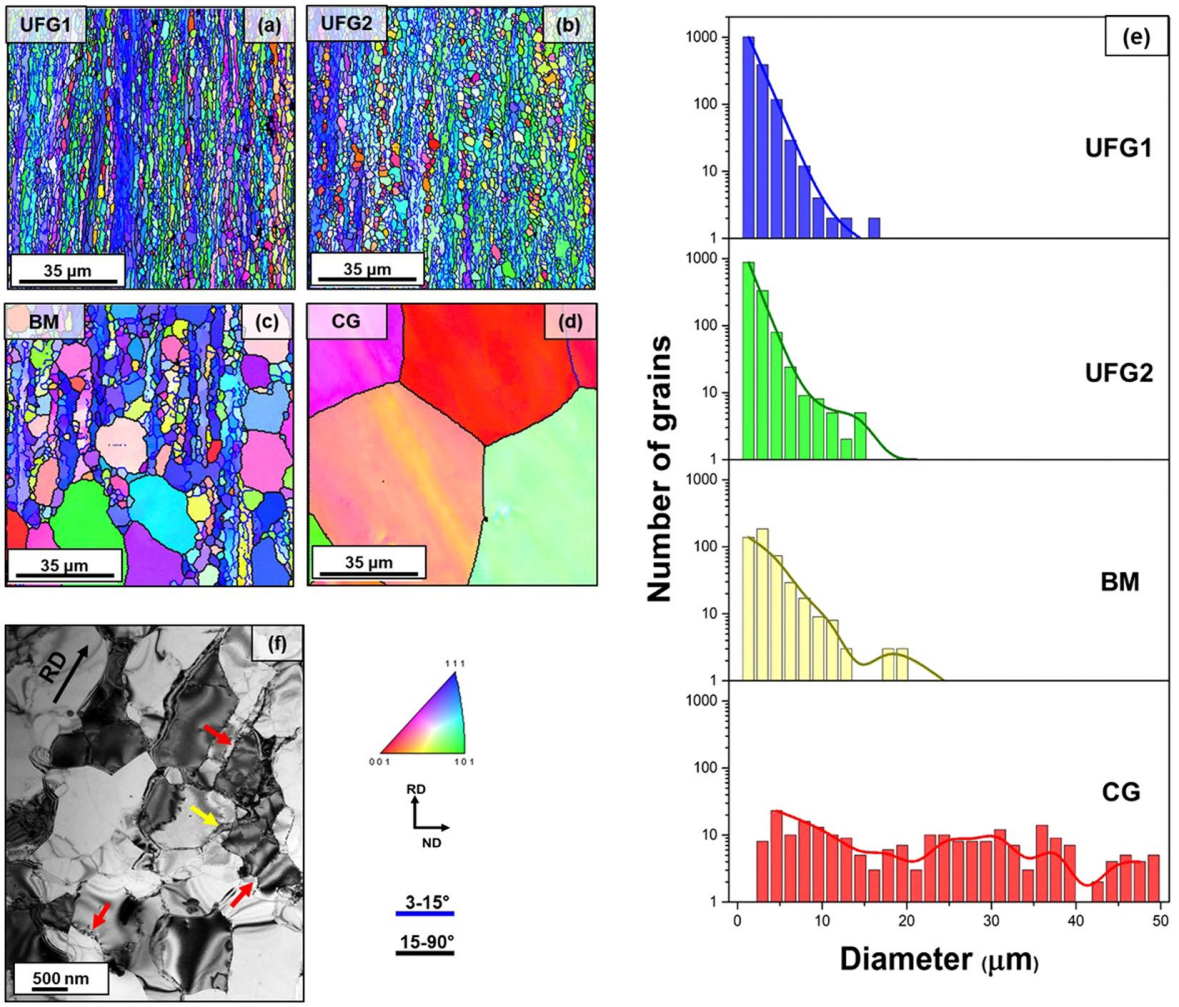
Microstructural analysis of the four different material engineered for the present investigation (**a–d**) EBSD map showing variation in grain size, distribution and morphologies of UFG1, UFG2, BM and CG material, (**e**) histogram showing the grain size distribution of each material, (**f**) TEM micrograph showing finer sub-granular details of UFG1 material. Elongated grain morphology and presence of residual dislocations in form of low angled grain boundaries (marked by red arrows) and dense dislocation walls (marked by yellow arrow) are observed in UFG1 material.

The EBSD inverse pole figure map obtained by analyzing the EBSD data points for the four samples is shown in Fig. 1(a–d). Grain sizes were measured using the linear intercept method, and the corresponding histograms are shown in Fig. 1(e). The average grain sizes, kernel average misorientation (KAM) angles, and percentage of high angled grain boundaries (HAGB) (i.e., with misorientation angle >15°) were calculated by using the TSL-OIM software, and the values of these parameters are presented in Table 1.

**Table 1:** Values of the key microstructural parameters calculated from EBSD datapoints.

Designation	Heat treatment (temperature, time)	Grain size (mean ± standard deviation)	Mean KAM angle	Percentage of HAGB
UFG1	200 °C, 0.5 h	1.23 ± 0.69 μm	1.07°	40.6%
UFG2	225 °C, 0.5 h	1.50 ± 0.81 μm	0.85°	49.2%
BM	250 °C, 0.5 h	3.05 ± 2.84 μm	0.67°	65.7%
CG	500 °C, 6 h	54.25 ± 46.56 μm	0.59°	70.8%

The EBSD maps of the samples clearly show the differences between all four engineered microstructures. UFG1 material contains grains with an average grain size of 1.23 μm with slightly elongated morphology. The grain’s substructures captured in TEM (Fig. 1(f)) reveal low angled dislocation subcells (marked by red arrow marks) and few dense dislocation walls (marked by yellow arrow). The microstructural parameters such as the relatively low percentage of HAGB (40%) and higher KAM angle (1.07) also correlate with the TEM and EBSD findings. This indicates that even after annealing at 200 °C for 30 minutes, the recovery process is incomplete, and the sample still preserves some of the signatures of CR. In UFG2 sample, a small increase of 9% in the percentage of HAGB is observed, which can be attributed to the completion of the recovery process and development of recrystallized, strain-free grains. This trend can also be correlated by the mean KAM angle (0.85°) which is less than 1° indicating full-recovery of the nanometric grains^25^. However, there is no significant grain growth, and the average grain size was found to be 1.5 μm which is close to the UFG domain. This slight increase is attributed to nucleation of near-micrometer sized recrytsallized grains. For the third sample (BM), the EBSD map shows a bimodal microstructure, where fully recrystallized coarse grains coexist along with ultrafine grains. Abnormal grain growth of specific grains has led to the development of such microstructure. The average size of the ultrafine grains is found to be 1.03 μm (volume fraction = 19.4%) and of the coarse grains is found to be 3.59 μm (volume fraction = 79.6%). The overall average grain size of the bimodal aggregate is found to be 3.05 μm. In this case, 68% of grains possesses HAGB, and the mean KAM angle is found to be 0.67°. The final sample (CG) contains consistently very coarse grains, and the average grain size is found to be 54.25 μm. More than 70% of the grains are HAGB, and the KAM angle is found to be 0.59°.

### Correlation between microstructure and size-effect during the uniaxial tensile loading

**Figure 2:**
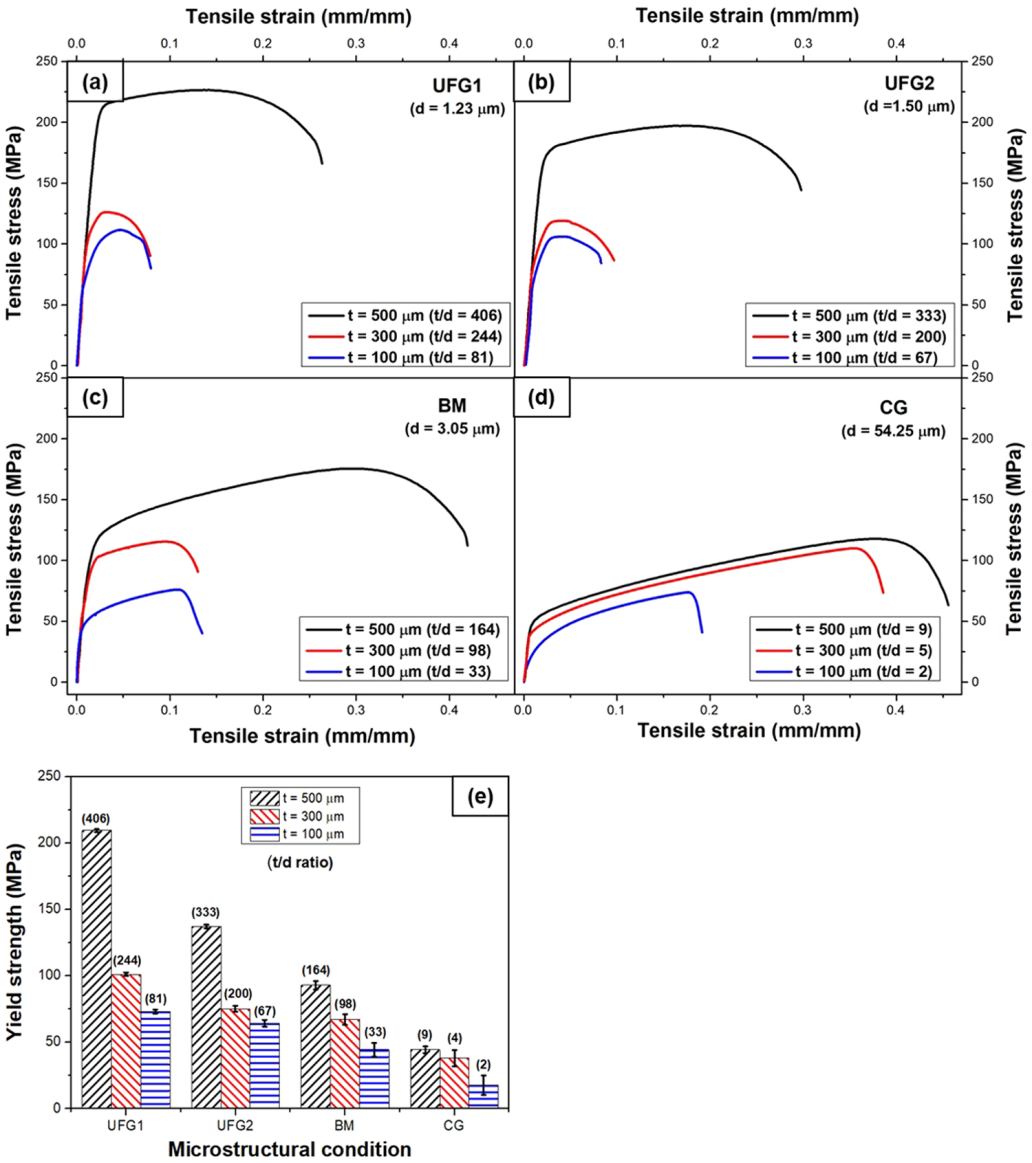
Tensile stress-strain curves for three different sample thickness for (**a**) UFG1, (**b**) UFG2, (**c**) BM and, (**d**) CG material. Bar graph showing the average and scatter values of yield strength for all four microstructures with respect to sample thickness. The values of t/d ratio corresponding to each sample are mentioned above the bars inside brackets.

The uniaxial tensile test was done on samples corresponding to all four microstructures and for each microstructural condition, three different sheet thicknesses (500, 300 and 100 μm) were tested. For each combination, five samples were tested, and true stress-strain curves corresponding to the average values are plotted for all these samples (Fig. 2(a–d)). For UFG1, UFG2 and, BM materials, there is a drastic change in the stress-strain response when the sample thickness is reduced from 500 μm to 300 and 100 μm. The slope of the curves is completely different from the 500 μm thick sample and the strength as well as the ductility of the materials are significantly reduced. This is quite different from trend of geometrical size effect observed in microforming due to increase in the volume fraction surface grain^26^. For UFG material, the volume fraction of surface grains is low and such effect is unlikely to occur. This suggests activation of an alternative deformation mechanism which results in the change in the tensile stress-strain response of the material. However, the tensile curves of the samples of 300 and 100 μm thickness for UFG materials are relatively similar. This indicates that the alternative deformation mechanism in UFG materials is comparatively insensitive to the thickness of the sample and is not influenced by size-effect. Similar trend is not found in BM material which shows that the BM material is partially affected by size effect. For the CG material, the tensile curves are progressively scaled down in terms of both strength and ductility with respect to the sheet thickness without a change in slope. This behavior is consistent with the trend observed for geometrical size effect in microforming^26^.

Based on the all the five stress-strain curves corresponding to each combination of microstructure and thickness, the average yield strength (at 0.2% proof stress) and their standard deviations have been plotted in a bar graph in Fig. 2(e). An increase in scatter of the yield strength is observed with the reduction in sheet thickness. The scatter is least in case of UFG1 microstructure and is remarkably high in case of CG material. The highest scatter is observed for CG material with 100 μm thickness. This behavior is also consistent with size effect related abnormalities during microforming. It is known that presence of few grains in the sample cross-section in case of CG material results in an increased anisotropy and scatter during micro-scale deformation.

### Qualitative evaluation of the micro-deep drawn components for different microstructures

**Figure 3:**
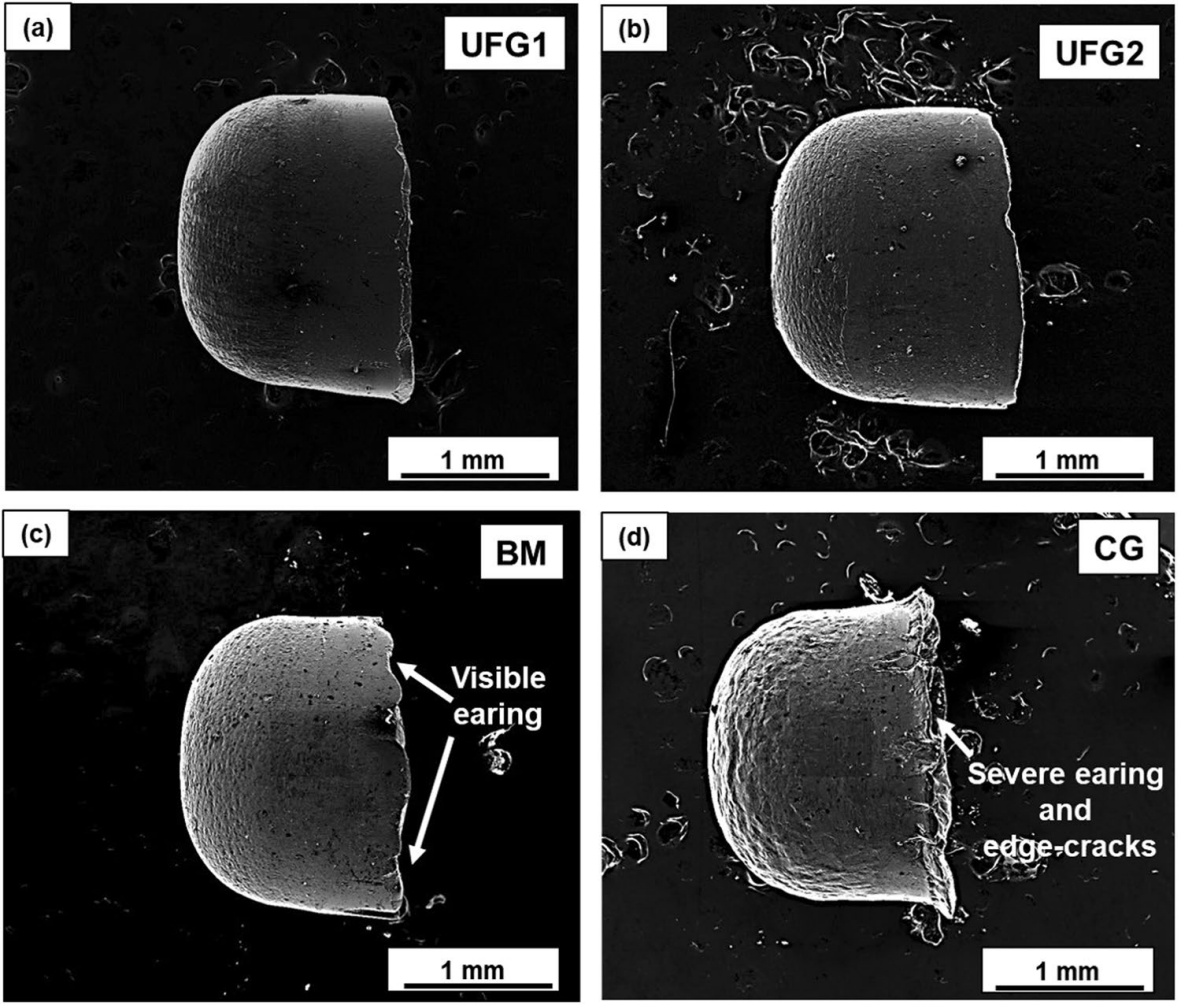
SEM images of the micro-cups for (**a**) UFG1 (**b**) UFG2 (**c**) BM and, (**d**) CG material. Defects such as edge-cracks and earing are clearly visible on the flange edge of the micro-cups corresponding to CG material.

To get greater insight into the microforming behavior, a micro-deep drawing setup was designed and developed. The sample of 250 μm thickness was chosen for this investigation, and the tool was designed based on this thickness (see Supplementary Figures). Micro-cups of 1.5 mm diameter were drawn from 3 mm circular blanks corresponding to all four microstructures. SEM images (side views) of the micro-cups have been presented in Fig. 3(a–d). The micro-cups of UFG materials has good form-accuracy and are devoid of undesirable defects. The slight asymmetry in the micro-cups could be attributed to the minor error in the concentricity of the blank and punch that occurs during positioning of the blank. The walls of the cups are free of cracks, wrinkles, and earing. On the other hand, for BM material few earing defects are visible under the SEM and no cracks and wrinkles are observed. However, for the CG material, the quality of micro-cup is significantly poorer. The micro-cup edges have severe earing defects, and cracks which have formed and propagated transversely towards the cup wall.

**Figure 4:**
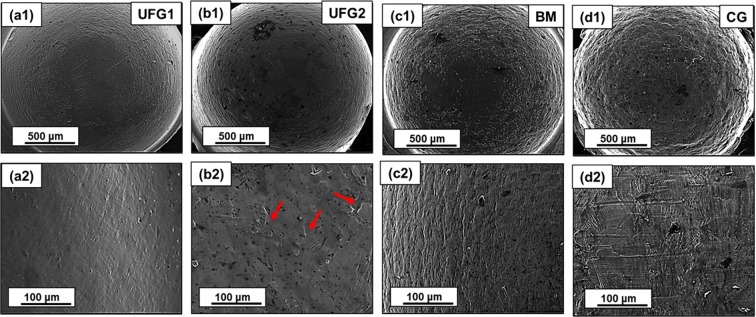
Low (**a1–d1**) and high (**a2–d2**) magnification SEM images showing the progressive degradation of the surface of the micro-cups (**a1,a2**) UFG1 has overall best surface quality (**b1,b2**) surface quality is also good for UFG2 except for the appearance of few minor bumps (red arrows) on the surface (**c1,c2**) circular ripple marks on the surface of BM micro-cups (**d1,d2**) significant undulation and crisscross striation marks on CG micro-cups.

The surface quality of the micro-cups also progressively deteriorates from UFG to CG materials. This is elaborately captured in the high magnification SEM images of the micro-cups’ bottom part (Fig. 4). Both in the case of UFG1 and UFG2 materials, the surface of the micro-cups appears very smooth. Very few small bumps are observed on the surface of UFG2 micro-cups (represented by red arrows Fig. 4(b)). On the micro-cups developed from BM material, distinctive circular wavy/ripple marks are observed. Finally, for CG material, the surface appears to have significantly degraded. Circular ripple marks appear more prominent compared to previous three materials. Apart from this, surface undulations and crisscross striations marks appear on this material.

### Cross-sectional strain measurement and postmortem EBSD analysis of the micro-cups

**Figure 5:**
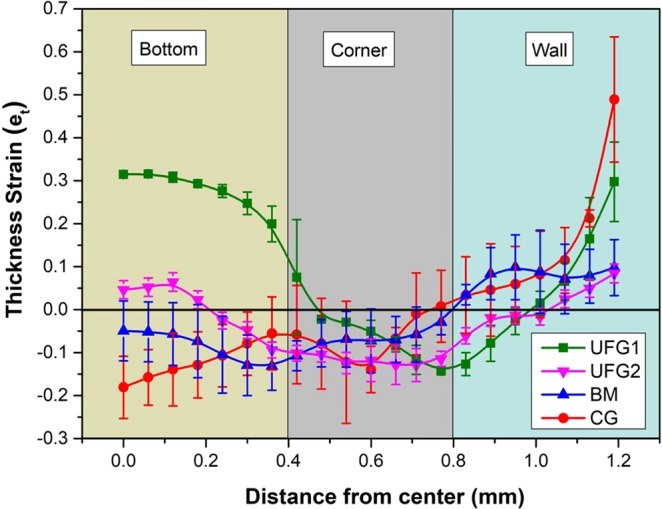
Variation of the thickness strain measured at cross section of the micro-cups plotted with respect to the distance from the center of the micro-cups. Note the large localization at the corner of UFG1 material. Scatter in measurement is high for BM and CG samples compared to the UFG materials.

Based on the cross-sectional SEM image of the micro-cups, strain at the thickness direction is measured at an interval of 50 μm along the sample cross-section. The thickness strain (e_t_ = (t_o_ − t_f_)/t_o_) is measured for 5 samples corresponding to each microstructure and the average and scatter values are plotted with respect to the distance from the center of the micro-cup (Fig. 5). Like macro sheet metal forming, strain localization at the specimen cross-section in microforming is considered detrimental and it is an indicator of reduced formability. Although form-accuracy and surface appearance of UFG1 are observed to be the best out of all four materials, cross-sectional observation reveals the occurrence of significant thinning at the corner of the micro-cups. This may be due to displacement of the materials from the corners of micro-cups. This behavior shows reduced formability of UFG1 material and implies greater susceptibility towards failure through localization and tearing. UFG2 and BM have relatively less variation in the thickness strain. Average strain thickness at some regions for UFG2 and BM material is close to zero which signifies minimal strain localization in the material. In the case of CG material, strain localization is also high especially in the wall and corners of the micro-cups. A large level thinning (localized strain) is observed in the wall section, which was not observed in previous cases. The scatter in the measured thickness strain is less for UFG1 and UFG2 but it increases significantly for BM and CG materials.

**Figure 6:**
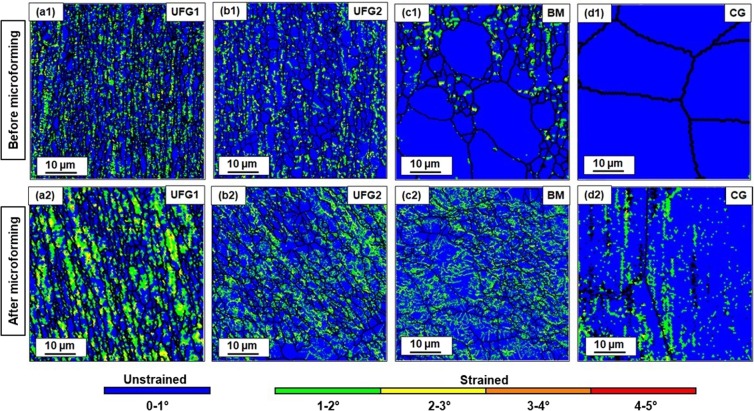
Cross-sectional KAM maps of the samples before (**a1–d1**) and after (**a2–d2**) microforming. The maps correspond to the corner region of the micro-cups. Bright green and yellow spots in case of UFG1 material indicates intense strain localization in the material.

To get a clearer understanding of the local plastic strain, the KAM values were mapped for all four engineered materials both before and after microforming (at the corner region of the micro-cups) and have been shown in Fig. 6. The corner region was chosen for KAM mapping because of its high susceptibility towards strain localization. Based on the data obtained from the KAM maps, the percentage of strained grains (with KAM angle >1**°**) and the mean KAM angles are calculated and given in Table 2.

**Table 2:** Mean KAM angles and percentage of strained grains before and after microforming.

Microstructure Condition	Mean KAM angle		Percentage of strained grains		
	Before microforming	After microforming	Before microforming	After microforming	Percentage increase in strained grain
UFG1	1.07°	1.21°	31.2	57.1	25.9
UFG2	0.85°	0.94°	12.3	27.6	15.3
BM	0.67°	0.91°	9.7	25.7	16
CG	0.59°	0.61°	4.3	12.2	7.9

The KAM angle is an indication of local plastic strain in the grains and the higher values of KAM angle suggest the presence of strained grains and greater concentration of geometrically necessary dislocations. The KAM map indicates extreme level of strain localization in the deformed grains for the UFG1 material. This observation correlates well with the large thinning at the corner of the micro-cup for the UFG1 material. For the remaining three materials, the intensity of local strains appears to be significantly lower. As compared to the intense local strain distribution in the grain interiors of UFG1 material, the KAM distribution appears to be less significant in the remaining three materials.

The percentage of strained grains (with mean KAM > 1°) after microforming is 57.1% in UFG1 material, which is approximately 26% greater than its value before microforming. On other hand, the percentage of strained grains increases by a factor of 15.3%, 16% and 7.9% after microforming for UFG2, BM and CG respectively. The presence of residual dislocations in the grain boundaries of the unrecovered grains and their elongated morphology in UFG1 microstructure are the prime reasons behind increased strain localization in this material.

**Figure 7:**
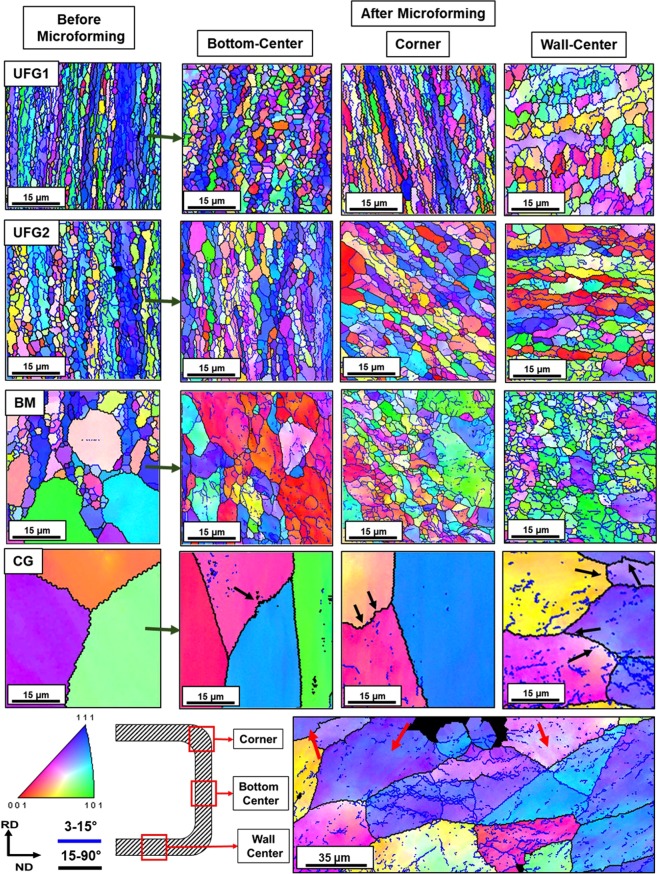
Cross-sectional EBSD images of the samples before microforming (left) and after microforming captured at the corner, bottom center and wall center region of the microcups. For UFG1 material, strain localiazation results in significant compression of the grains at corner. Stress-induced grain growth in UFG2 due to uniform grain migration and rotation is observed which synergetically accomodates the complex microforming strain paths. For BM dual mode of deformation is observed. For CG material, size effect reduces the propensity of cross-slip (red arrows) and results in grain bulging (black arrows). See Supplemetary Figures for large scale EBSD maps of CG material captured at all three locations.

In addition to the KAM mapping, EBSD inverse pole figure were also mapped on the cross-section of the micro-cups at three locations: center of the bottom part, corner and, center of the wall of the micro-cups. The EBSD map corresponding to UFG1 shows very thin and elongated grains at the corner, which correlates well with the large level of thinning observed in this material. At the wall, the level of transverse elongation of the grains are observed to be prominent, and significant grain coarsening was also observed. For UFG2, the EBSD map shows a synergetic movement of grains which corresponds well with the complex deep drawing strain path and a uniform grain coarsening at all three regions of the micro-cup. In the case of BM material, smaller grains have undergone deformation via GBMP mechanisms, while in the interiors of larger grains a network of sub-grains is observed. The sub-grain formation is also observed in the case of CG material. Surprisingly, compared to BM material the level of sub-grains formation is much lower in the case of CG material, which is in contradiction to the fact that subcell formation is energetically favorable for larger grains. The inability to slip in these materials signifies reduced plasticity due to the influence of size-effect. Grain bulging and buckling are also observed in some grains of CG material (marked by arrows). EBSD map taken over a larger area shows a significant difference between the sub-grain density inside the surface grains (marked by red arrows in Fig. 7) and core grains. Large scale EBSD map taken at corner and bottom part of the micro-cups drawn from CG material is shown in the Supplementary Figures.

## Discussion

Slip based macroscopic deformation mechanism of conventional coarse-grained materials is the congregation of millions of microscopic deformation activities occurring in sub-granular level. It is a well-understood phenomenon which involves the development of dislocation slip predominately from the grain boundaries, dislocation slip motion along the grain interiors, and finally, its accumulation leading to localization and subsequent failure. For high SFE material such as aluminum, slip based deformation mechanism such as easy-glide and cross-slip are proactive. Cross-slip leads to formation of extensive networks of sub-grains in the grain interiors, which can be captured via electron microscope. In the case of UFG materials, the volume fraction of grain boundaries is much larger compared to coarse-grained materials. This reduces the mean free path for dislocation motion and constrains the dislocation multiplication. Therefore, deformation via conventional mode of plasticity leads to rapid strain localization and limited uniform ductility^27^. However, at a certain sample length scale, instead of the conventional mode of plasticity, grain boundary-mediated plasticity (GBMP) such as grain sliding/migration, rotation and creep are activated, and they significantly contribute to the overall plastic deformation of nanostructured and UFG materials^28–31^. Literature suggests that grain migration is the dominant mode of GBMP and a prime contributor to the deformation strain^32^. Grain rotation is a secondary effect and is triggered to accommodate the grain migration phenomenon. The mechanism of grain rotation involves cross-grain dislocation glide and/or climb, and is only significant during the initial stages of the deformation. These GBMP phenomena contribute to stress-assisted grain growth due to migration, rotation and coalescence of the grains in a preferred direction in order to accommodate the deformation strain paths^33^. Grain creep are found to be insignificant during room temperature deformation of material and during medium to high deformation strain rates. Therefore, this phenomenon is not expected to be active in the present case. Researchers have captured GBMP phenomena by *in-situ* TEM micrography. However, postmortem analysis of the stress-assisted grain growth can also be used to provide intutive evidence for the occurrence GBMP phenomena such as grain migration and rotation. Due to the unstable nature of the grain boundaries in UFG material, GBMP is accompanied by a rapid rate of stress-assisted grain growth which results in abnormal coarsening of grains to micrometer scale^34–36^. At initial stage, stress-assisted grain growth promotes plastic deformation. However, when the grains grow beyond the critical size, slip-dominated plasticity may be reactivated, and size effect may interfere the microscale plastic deformation. Unlike the slip-based deformation, plastic deformation via GBMP is unaffected by size-effect associated with microforming.

In the present case, the transition in the tensile stress-strain curves (Fig. 2(a,b)) observed for the UFG material in the thickness domains of 300 and 100 μm is the first indicator of the occurrence of GBMP. Critical stress required to activate GBMP is lower than slip-dominated plastic deformation lowering of the yield strength of the materials. Reactivation of slip due to concurrent grain growth results in strain localization and subsequent failure in UFG materials. This results in a lower uniform ductility in the corresponding tensile curves. Since this mechanism is independent of size-effect, the variation in the tensile curves between 300 and 100 μm samples is negligible.

The postmortem EBSD analysis of the micro-cups gives visual evidence of the stress-assisted grain growth after microforming (Fig. 7), which indicates activation of GBMP phenomena such as grain migration and rotation. It is known that UFG1 possesses slightly elongated grains and possesses residual dislocation in the grain boundaries (due to partial recovery). This obstructs the effective grain migration and rotation, accelerates strain localization and results in the thinning at the corner of the micro-cups. On the other hand, UFG2 with ultrafine, equiaxed and obstruction-free (residual dislocation) grains undergoes uniform grain migration and rotation throughout the micro-cup due to activation and effective promotion of GBMP phenomenon. This is in good synergy with the complex micro-deep drawing strain paths throughout the micro-cup, resulting in good surface morphology and uniformity in the sample cross-section. Therefore, for effective grain migration and rotation in the material, not only the grain size is the critical factor, but also the nature of the grain boundaries, misorientation angle and morphology are also equally important.

In case of BM material, a mixed mode of deformation is found: (i) grain migration and rotation in smaller grain and (ii) activation of cross-slip in coarser grains resulting in extensive sub-grain formation in their interiors. The coarser grain reduces the propensity of GBMP resulting in partial loss of its microformability. Surprisingly, the coarse grains in BM material undergo an appreciable amount of cross-slip, without getting adversely affected by size-effect. In case of CG material, due to the presence of uniform coarse grains, migration and rotation of grains are not observed, and only slip-dominated plasticity prevails in the material. However, the cross-slip activities are much lesser in CG material compared to coarse grains of the BM material. This can be correlated from the EBSD map showing significantly less density of sub-grain in the grain interiors of CG materials. This is in agreement with the observation that cross-slip activities are delayed during miniaturization in case of high SFE materials due to an increase in the volume fraction of the surface grains^37^. This is evident in the cross-sectional EBSD micrographs of CG material taken over a larger area, which shows relatively lower density of sub-grains in the surface grains compared to the core grains (red arrows in Fig. 7). Limited cross-slip in CG material leads to generation of intragranular shear strain which results in the observed grain boundary bowing and bulging (black arrows in Fig. 7). The strain incompatibility in the surface grains and core grain interface leads to non-uniform microscopic protuberation of the surface grain in the less constrained normal direction. Aggregation of these microscopic projection results in the poor surface quality of the micro-cups of CG material (Fig. 4).

## Conclusions

This comprehensive investigation reveals that due to the high density of grain boundaries in UFG materials, grain boundary-mediated plasticity phenomena such as grain migration and rotation are activated at the microscale level. This results in improved microformability, uniform granular deformation, strain delocalization and minimization of size-effect related abnormalities associated traditionally with microforming. For grain migration and rotation to occur efficiently, the UFG microstructure should be equiaxed, high-angled and should be devoid of obstructive dislocations. Micro-cups developed from this material possess excellent surface quality, good form-accuracy and minimal process scatter. On the other hand, the coarse-grained material undergoes microscale deformation via traditional slip and cross-slip based mechanism. This makes the material susceptible to size-effect related abnormalities and reduces its microformability.

## Supplementary information

Supplementary Figures
