# Supplementary material for: Achieving excellent microformability in aluminum by engineering a unique ultrafine-grained microstructure
Source: Sci Rep. 2019 Jul 23;9:10683. doi: 10.1038/s41598-019-46957-4 (PMC6650420; doi:10.1038/s41598-019-46957-4)
Supplement: Supplementary file 1 — Supplementary Figures [file 41598_2019_46957_MOESM1_ESM.docx]

**Achieving excellent microformability in aluminum by engineering a unique ultrafine-grained microstructure**

A. Dhal^1^, S.K. Panigrahi^1*^, M.S. Shunmugam^1^

^1^Manufacturing Engineering Section

Department of Mechanical Engineering

Indian Institute of Technology Madras, Chennai- 600036, India


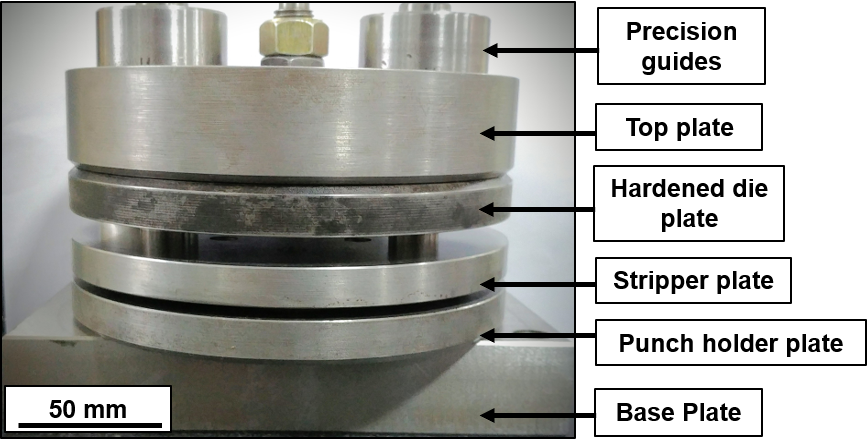


**Supplementary Figure 1.** Micro-deep drawing tool used in the present investigation


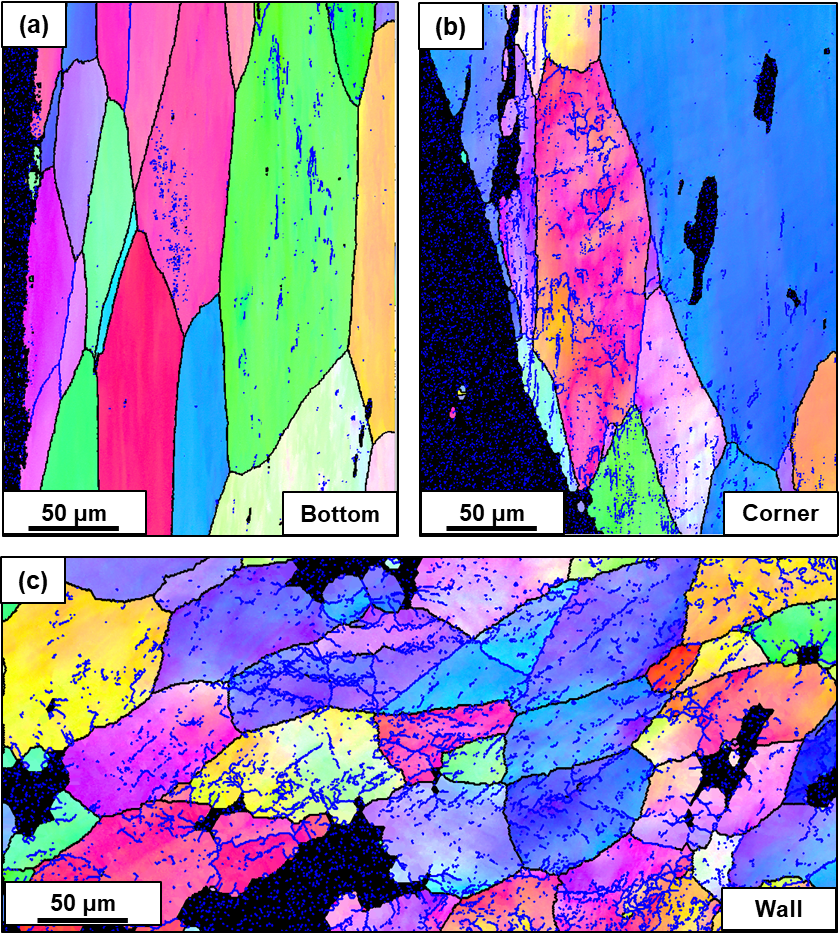


**Supplementary Figure 2.** Large scale EBSD maps of CG material captured at (a) bottom, (b) corner and (c) wall region of the micro-cup cross-section.
